# Database of ab initio L-edge X-ray absorption near edge structure

**DOI:** 10.1038/s41597-021-00936-5

**Published:** 2021-06-11

**Authors:** Yiming Chen, Chi Chen, Chen Zheng, Shyam Dwaraknath, Matthew K. Horton, Jordi Cabana, John Rehr, John Vinson, Alan Dozier, Joshua J. Kas, Kristin A. Persson, Shyue Ping Ong

**Affiliations:** 1 Department of Nanoengineering, University of California San Diego, La Jolla, CA 92093 USA; 2 Materials Science Division, Lawrence Berkeley National Laboratory, Berkeley, CA 94720 USA; 3 Department of Chemistry, University of Illinois at Chicago, Chicago, IL 60607 USA; 4 Department of Physics, University of Washington, Seattle, WA 98195 USA; 5 Material Measurement Laboratory, National Institute of Standards and Technology, Gaithersburg, MD 20899 USA; 6 Health Effects Laboratory Division, National Institute for Occupational Safety and Health, Centers for Disease Control, Cincinnati, OH 45226 USA; 7 Department of Materials Science and Engineering, University of California Berkeley, Berkeley, CA 94720 USA; 8 Molecular Foundry, Lawrence Berkeley National Laboratory, Berkeley, CA 94720 USA

**Keywords:** Computational methods, Characterization and analytical techniques

## Abstract

The L-edge X-ray Absorption Near Edge Structure (XANES) is widely used in the characterization of transition metal compounds. Here, we report the development of a database of computed L-edge XANES using the multiple scattering theory-based FEFF9 code. The initial release of the database contains more than 140,000 L-edge spectra for more than 22,000 structures generated using a high-throughput computational workflow. The data is disseminated through the Materials Project and addresses a critical need for L-edge XANES spectra among the research community.

## Abstract (summary)

**Table:** 

Measurement(s)	near-edge X-ray absorption fine structure spectroscopy • L-edge XANES
Technology Type(s)	X-ray absorption spectroscopy

Machine-accessible metadata file describing the reported data: 10.6084/m9.figshare.14233055

## Background & Summary

X-ray absorption spectroscopy (XAS) is a robust and valuable characterization technique for accurate identifications of atomic local environments^1^, oxidation states^2^, and electronic structures^3^, etc. In XAS, core electrons are excited after the absorption of X-ray photons. The edge names are based on the principal quantum number of the electrons excited: K for 1 s, L for 2 s or 2p,etc. The XAS can be further divided into the X-ray absorption near edge structure (XANES) and the extended X-ray absorption fine structure (EXAFS) by energy range. The XANES region often has stronger signal and can be directly correlated with atomic local environments and oxidation states of the absorbing atoms by comparing to known reference spectra. Unfortunately, the availability of reference XANES data is typically less than other X-ray based characterization techniques due to its reliance on high-energy synchrotron sources.

Researchers confront such deficiencies by gathering data from the community to construct calibrated databases^4^. For example, the EELS database (EELS DB)^5^ hosted by the European Microscopy Society contains several hundred spectra covering 35 elements. More recently, Diamond Light Source has integrated data processing and collection to improve traceability^6^. In addition, researchers are establishing international collaborations to further enhance XAS data distribution and sharing^6,7^.

As a complementary alternative to experimental XAS databases, computational XAS databases are attracting increasing interest. Codes based on multiple scattering^8,9^, multiplet^10^ and Bethe-Salpeter equation^11–14^ methodologies are commonly used in computing the XAS. Unlike semi-empirical multiplet calculations that impose strong restrictions on symmetry options, *ab initio* techniques based on the Bethe-Salpeter equation and multiple scattering are able to account for deviations from the idealized coordination environment around the absorbing atom. Previously, the present authors have used the FEFF9 multiple scattering code^8^ to generate the world’s largest computational database of K-edge XANES spectra, the XASdb^15,16^. The XASdb currently hosts more than 500,000 K-edge XANES spectra for more than 51,000 materials, while most experimental databases only contain several hundred. The XASdb has already been extensively used in many works, including the development of machine learning models to accelerate the interpretation of XAS. For example, Zheng *et al*.^17^ applied random forest models to predict coordination environment labels using XANES spectra from the XASdb. Andrejevic *et al*.^18^ demonstrated that neutral network-based models are capable of classifying topological materials solely from XANES spectra by training the models on XASdb. Both works delivered >80% average accuracy among dozens of absorbing elements. Specifically for 3d transition metal elements, researchers have adopted related data from XASdb to predict coordination numbers, Bader charges, mean nearest neighbor distances^19^ and local coordination environments^20^.

In this work, we further extend the XASdb by developing a database of L-edge XANES using the FEFF9^8^ code. The L-edge is especially useful in the study of transition metal elements where the K-edge energies are too high for facile measurements. For instance, the L-edge XANES has been routinely used to investigate how strain, ligand, and particle size affect the performance of catalysts^21^. The L-edge XANES can be further divided into L_1_ and L_2,3_ edges. The L_1_-edge representing quadrupole transitions is typically broadened, weak and lacks significant information for interpretation^22^. Thus our focus is on the L_2,3_-edge derived from intense dipole-allowed transitions. Our L-edge XANES database covers around 140,000 L_2,3_-edge XANES for more than 22,000 structures. It has been integrated as part of the XASdb hosted on the Materials Project^23^ website for individual spectrum visualization and download. A complete set of L-edge data can be downloaded separately in JSON format. This L-edge dataset serves as a reliable repository of reference spectra and enables machine learning applications using L-edge data.

## Methods

### Theory

In the perspective of computational XAS, Fermi’s golden rule governs the transition probability for a given initial state $| i\rangle$ and final state $| f\rangle$. This transition rate is proportional to X-ray absorption coefficient, and can be expressed in terms of the wavefunction as follows:

1 $$\mu (E)\propto \sum _{f}| \langle {\psi }_{i}| A(r)\cdot p{| {\psi }_{f}\rangle | }^{2}\delta (E-{E}_{f})$$

where ${\psi }_{i}$ and ${\psi }_{f}$ are the initial and final eigenstates, and *A*(*r*) · *p* accounts for the coupling of X-ray field. The L_2,3_-edge XANES is due to 2p-to-*n*d transitions.

If written in terms of the one-particle Green’s function, *µ* can be calculated in a more efficient way using the following equation:

2 $$\mu (E)=-\frac{1}{\pi }Im\langle i| \widehat{\varepsilon }\cdot r{\prime} G(r,r;E)\widehat{\varepsilon }\cdot r| i\rangle $$

where the $\widehat{\varepsilon }\cdot r$ is the interaction operator in dipole approximation. $G(r,r;E)$ can be further divided into the contribution from the central atom, ${G}^{c}$ and the contribution from the remaining scatters ${G}^{sc}$ (see ref. ^8^ and references therein). This theoretical formalism is implemented in the *ab-initio* real-space multiple-scattering XAS code FEFF9 for general calculations of X-ray spectra throughout the periodic table^8^. For a more thorough background in the theory, we direct the interested readers to the review paper by Rehr *et al*.^24^.

### High-throughput workflow

**Fig. 1:**
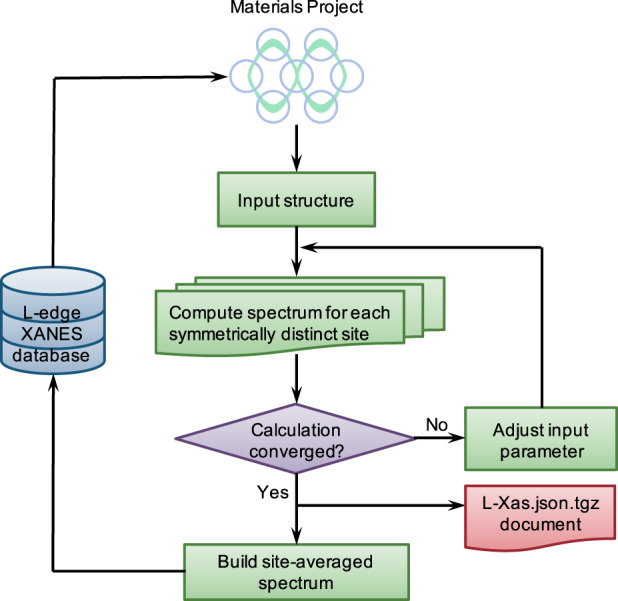
Schematic diagram of high-throughput workflow for L-edge XANES computation and data processing.

A high-throughput workflow for L-edge calculations was developed using pymatgen^25^, FireWorks^26^ and atomate^27^, as shown in Fig. 1.

The initial input structure is obtained from relaxed structures in the Materials Project^23^ database. For each symmetrically distinct site within the structure, a site-wise spectrum is computed with automatic convergence checks and error recovery using the custodian package^28^. Then the raw site-wise spectra are averaged to element-wise spectra based on site multiplicity and inserted into the XASdb, which can be accessed via the Materials Project website.

At the present moment, only structures in the Materials Project that originated from the Inorganic Crystal Structure Database (ICSD)^29^, i.e., likely to have been experimentally synthesized previously, have been calculated. Further, only elements with atomic number larger than 20 are included because the characterization of lighter elements are typically based on the K-edge XANES.

### Data analysis

The cosine similarity is used to evaluate the similarity between spectra quantitatively^30^. It is defined in Eq. 3 as:

3 $${\rm{Cosine}}\;{\rm{similarity}}\;{\rm{(A,}}\,{\rm{B)}}=\frac{{\overrightarrow{I}}_{A}\cdot {\overrightarrow{I}}_{B}}{\left\Vert {\overrightarrow{I}}_{A}\right\Vert \left\Vert {\overrightarrow{I}}_{B}\right\Vert }$$

where ${\overrightarrow{I}}_{A}$ and ${\overrightarrow{I}}_{B}$ represent intensity vectors for the two spectra. It was computed using the scikit-learn^31^ package.

## Data Records

**Fig. 2:**
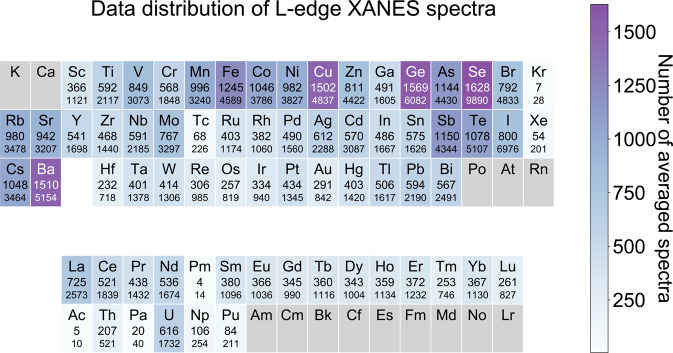
Data distribution for the L-edge XANES database. The upper and lower numbers for each element correspond to the number of site-averaged (upper) and site-wise count (lower), respectively. One site-averaged count refers to a crystal structure where both its corresponding L_2_ and L_3_-edge site-wise XANES spectra are complete. One site-wise count refers to either L_2_ or L_3_ spectrum for a single absorbing site. The figure is colored in terms of the site-averaged count.

To date, approximately 140,000 site-wise L_2_ and L_3_ spectra have been computed, which correspond to 40,000 site-averaged L_2,3_-edge XANES spectra for unique crystals. The data distribution for the L-edge XANES dataset is shown in Fig. 2.

### Data visualization

The L-edge data were integrated with the previous K-edge data for a comprehensive display of XAS spectra on Materials Project website (https://materialsproject.org/xas). The website allows users to compare the XANES spectra interactively for K, L_2,3_, L_2_ and L_3_-edge XANES spectra although L_2,3_ functionality is only supported for absorbing elements with 21 ≤ Z ≤ 30. Users can also apply Gaussian smoothing to the spectra on-the-fly to account for instrumental broadening.

### Data download

The dataset can be downloaded in two ways. For users interested in specific chemical systems (e.g., searching for reference spectra within Ni-O system), an API query (https://api.materialsproject.org/docs#/XAS) through the representational state transfer (REST) endpoint interfaced with the XASdb is recommended. The data obtained through API are site-averaged spectra, which can be directly compared to experimental spectra. The second method is to download a L-XAS.json.tgz file for a complete set of site-wise L-edge data through figshare (10.6084/m9.figshare.12824513.v1)^32^. The complete dataset is appropriate for data-intensive applications such as machine learning. The detailed data structure is shown in Table 1.

**Table 1:** Data structure for individual entry in L-XAS.json.tgz.

Property	Description
xas-id	Unique id for each site-wise spectrum in the form of mpid-siteindex-spectrumtype-edge (e.g., mp-24850-1-XANES-L3)
edge	Absorption edge (L_2_ or L_3_)
mp-id	Unique materials project ID (e.g., mp-24850)
structure	Pymatgen Structure object in JSON format
absorbing atom	Site index in structure (e.g., 0)
input parameters	FEFF9 input parameters in dictionary form
spectrum	Array of shape (2, 100) where the first row is energies in eV and the second row is absorption coefficients in arbitrary unit

## Technical Validation

We performed validation of FEFF9’s input parameters based on a comparison between computational and experimental spectra. Unlike the previous K-edge database works^15,16^ that covered a more thorough benchmark on FEFF9 input parameters, only critical input parameters that shape the L-edge spectrum, such as the cluster radius in self-consistent field (SCF) calculations and the core-hole treatment, are discussed here. A more detailed discussion about the full multiple scattering (FMS) calculations and the choice of exchange correlation potential, which are kept the same for the L-edge calculations, can be found in previous works on the K-edge calculations^15,16^ for interested readers. While more advanced treatments such as Debye-Waller factors can be performed using FEFF9, we did not benchmark them in this high-throughput work. A total of 18 experimental reference spectra were collected and listed in Table 2. We included diverse absorbing species (both 3d transition metal and heavy 4 f absorbing species) and various local environment to unveil a convergent set of inputs.

**Table 2:** Details of 18 experimental reference spectra.

Formula	Absorbing species	Materials id	Space group	Reference
TiO_2_	Ti	mp-2657	P4_2_/mnm	^36^
V_2_O_5_	V	mp-754670	Pmmn	^37^
MgCr_2_O_4_	Cr	mp-19202	Fd$\bar{3}$m	^38^
Cr_2_O_3_	Cr	mp-19399	R$\bar{3}$c	^39^
LiMn_2_O_4_	Mn	mp-25015	Fd$\bar{3}$m	^40^
MgMn_2_O_4_	Mn	mp-32006	I4_1_/amd	^40^
MnO_2_	Mn	mp-714975	P4_2_/mnm	^41^
FeF_3_	Fe	mp-22398	R$\bar{3}$c	^42^
LiFePO_4_	Fe	mp-19017	Pnma	^43^
LiCoPO_4_	Co	mp-18915	Pnma	^44^
LiCoO_2_	Co	mp-24850	R$\bar{3}$c	^45^
CoF_2_	Co	mp-556520	P4_2_/mnm	^46^
NiF_2_	Ni	mp-559798	P4_2_/mnm	^46^
KNiF_3_	Ni	mp-560976	Pm$\bar{3}$m	^47^
Cu	Cu	mp-30	Fm$\bar{3}$m	^48^
CuO	Cu	mp-1692	P4_2_/mmc	^48^
Pt	Pt	mp-126	Fm$\bar{3}$m	^49^
CeO_2_	Ce	mp-20194	Fm$\bar{3}$m	^50^

**Fig. 3:**
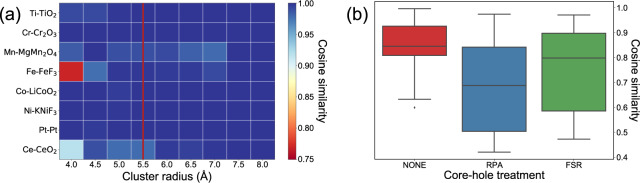
Benchmarking results for (**a**) cluster radius for SCF, and (**b**) core-hole treatment parameters for FEFF9 L-edge XANES calculations. When benchmarking cluster radius for each chemical system, the cosine similarities are computed with respect to the spectrum that is calculated with a cluster radius of 8 Å. A comparison between L_2,3_-edge XANES is conducted for all absorbing elements except the heavy 5f elements Ce and Pt. For Ce and Pt, only the L_3_-edge XANES are compared because of large energy separation between the L_2_ and L_3_ peaks. For core-hole treatment, the cosine similarities are calculated between the computed spectra and experimental spectra for all chemical systems listed in Table 2.

The cluster radius in SCF calculations determines the number of atoms to be included in the coordination shell. A sufficiently large cluster radius is necessary to account for all scattering effects but an excessively large one will cause an increase in computation cost and an overestimation of multiple scattering from long-distance atoms^33^. From Fig. 3, we found that a cluster radius of 5.5 Å was sufficient to converge the L-edge spectra to a cosine similarity of 0.98 compared to a cluster radius of 8 Å.

Another important input parameter to FEFF9 calculations is the core-hole treatment. When the core electron is excited by X-ray photons, it creates a photon-electron or core-hole pair. Among all three core-hole treatments supported by FEFF9, i.e., the final state rule (*FSR*), the random phase approximation (*RPA*) and no core-hole (*NONE*), it was found that calculated spectra without core-hole treatment resulted in the best agreement (highest cosine similarity) with experiments, as shown in Fig. 3. *FSR* sometimes breaks down for L-shell calculations while *RPA* can cause an irregular shake-up in the post-edge region of the XANES spectrum. Further, the more mobile electrons at outer shells (e.g., 2p vs. 1 s electrons) lead to stronger screening of the core-hole effect than can be accounted for with *FSR* or *RPA*.

**Fig. 4:**
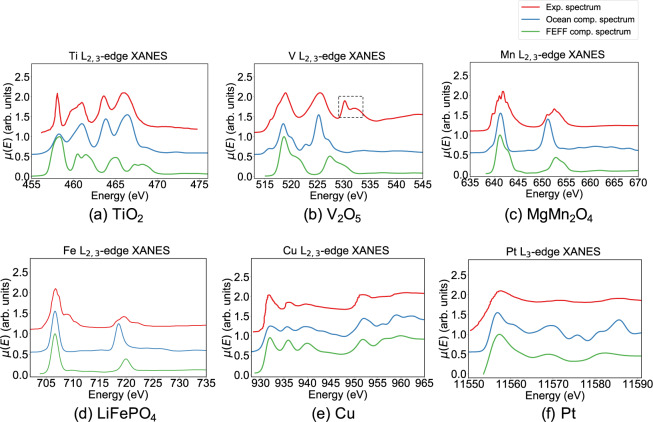
Comparison between experimental spectra, ocean computed spectra and FEFF9 computed spectra. The first and second major peaks are the L_3_ and L_2_ peak, respectively. The third peak in experimental V L-edge spectrum in *α*-V_2_O_5_, indicated by the black dashed rectangle, is contributed by the oxygen K-edge, which is not accounted for in the computed spectra. The details of experimental data can be found in Table 2. Spectra are shifted vertically and normalized to maximum intensity for ease of visualization.

In Fig. 4, the spectra calculated using the FEFF9 converged input parameters are compared against experimental spectra as well as spectra computed using ocean^11,12^, an alternative software based on the Bethe-Salpeter equation for XANES calculation. A horizontal shift to align the L_3_ peak and a Gaussian broadening with full width at half maximum (FWHM) of 1.2 eV were applied to the computed spectra to account for the less accurate determination of Fermi level and the instrumental broadening, respectively. In general, the FEFF9 spectra are in relatively good agreement with experimental measurements and the ocean computed spectra in terms of relative peak positions and peak intensities. For Ti in rutile TiO_2_ and V in *α*-V_2_O_5_, the L_2_ peak intensities and the edge separation computed by ocean are in better agreement with experiments than FEFF9, which can be explained by the inclusion of spin-orbit coupling during ocean calculation. However, the significantly higher computational costs and poorer scalability of ocean calculations make it less suitable for high-throughput generation of spectra.

**Fig. 5:**
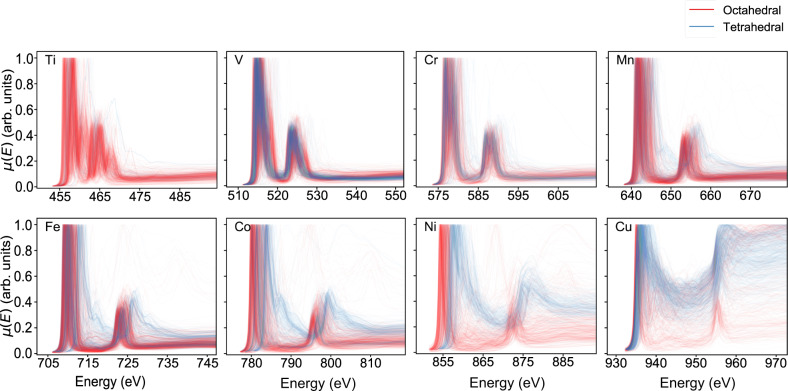
L_2,3_-edge XANES spectra for 3d transition metal elements. The colors represent the local environments around absorbing atoms where red color refers to octahedral environment and blue is for tetrahedral environment. Spectra are normalized to maximum intensity for ease of visualization.

The L_2,3_-edge XANES spectra for eight 3d transition metal elements in various crystals are plotted in Fig. 5. These elements are predominantly in the octahedral and tetrahedral local environments^34^. Except for Ti and Mn where the octahedral environment dominates, the rest of the 3d transition metals have a more even distribution of octahedral and tetrahedral local environment. The L_3_/L_2_ branching ratio, defined as the ratio of integrated intensities for L_3_ and L_2_ peaks, is vital to the spin states because this property is proportional to the number of paired electrons. In theory, the branching ratio for an absorbing atom in tetrahedral environment is smaller than that in octahedral environment. The crystal field splitting for tetrahedral environment is smaller than that of octahedral environment, leading to a preference for high-spin states over low-spin states in the tetrahedral environment. This is consistent with the observation that the L_2_ peak intensities for 3d transition metals such as Mn, Fe, Co, Ni and Cu in tetrahedral environment are significantly larger than in the octahedral environment. Another important finding comes from the decrease of white-line intensities. The white line refers to the intense absorption in the rising edge region of a XANES spectrum. Its intensity is positively related to the number of unoccupied d states. With increasing number of valence states, the white line intensities continue to decrease along with filling of d orbitals. While most FEFF spectra trends are consistent with theory, we observed that a small number of spectra (53 out of around 140,000 site-wise spectra) possess unphysical negative intensities. This could be caused by the instability when directly solving the complex Green’s function, which leads to small negative spectra weight since the phase might be wrong. Another possible explanation lies in the numerical precision differences between the atomic background and the scattering contributions, which are calculated separately. While extra caution is needed, it is up to users and use cases to determine how to treat those spectra with negative intensities.

## Usage Notes

Each entry in the database contains site-specific or site-averaged L-edge XANES (as well as K-edge XANES), together with physical and chemical properties such as the elemental oxidation states and coordination environments. These data can be used as references for comparison with experimental spectra to identify the properties of species present or local environment when using a very fine electron energy loss spectroscopy (EELS) probe. While there are discrepancies between the computed and experimental spectra for certain elements (especially the early transition metals), this can be mitigated through computations with other high-level codes such as ocean for select systems.

Another potential usage of this dataset is in the development of machine learning (ML) models to accelerate spectra interpretation. ML models such as random forest^17,19^ and neural networks^18,20^ have been developed using our previously-constructed K-edge XANES database, with accuracies exceeding 80% having been achieved in classifying the local environment. Potentially, similar or more refined ML models, especially for transition metal systems, can be developed using both the K-edge and L-edge XANES. For instance, the branching ratio in L_2,3_-edge XANES can be used to distinguish between different spin states even in the same complexes^35^.

In summary, a large dataset covering 140,000 L_2,3_-edge XANES spectra are made open to the public after an in-depth benchmark, complementing the world’s largest computational XAS database. Our benchmark results indicate that multiple scattering codes such as FEFF9 can achieve comparable accuracy when compared to experimental data. We anticipate this work to benefit the whole XAS community through well-curated data and elaborated dissemination.

## Data Availability

The workflow for FEFF9 calculation including input generation, output parsing and workflow management is available in open-source materials science packages pymatgen^25^, FireWorks^26^ and atomate^27^. The error handler for automatic error detection and recovery can be found in custodian^28^.
